# Short-term air pollution exposure associated with death from kidney diseases: a nationwide time-stratified case-crossover study in China from 2015 to 2019

**DOI:** 10.1186/s12916-023-02734-9

**Published:** 2023-01-24

**Authors:** Miao Cai, Jing Wei, Shiyu Zhang, Wei Liu, Lijun Wang, Zhengmin Qian, Hualiang Lin, Echu Liu, Stephen Edward McMillin, Yu Cao, Peng Yin

**Affiliations:** 1 Department of Epidemiology, School of Public Health, Sun Yat-Sen University, Guangzhou, 510080 Guangdong China; 2 Department of Atmospheric and Oceanic Science, Earth System Science Interdisciplinary Center, University of Maryland, College Park, MD 20740 USA; 3 National Center for Chronic and Noncommunicable Disease Control and Prevention, Chinese Center for Disease Control and Prevention, Beijing, 100050 China; 4 Department of Epidemiology and Biostatistics, College for Public Health and Social Justice, Saint Louis University, St. Louis, 63103 USA; 5 Department of Health Management and Policy, College for Public Health and Social Justice, Saint Louis University, St. Louis, MO 63103 USA; 6 School of Social Work, College for Public Health and Social Justice, Saint Louis University, St. Louis, MO 63103 USA; 7 Information Center, Chinese Center for Disease Control and Prevention, Beijing, 102206 China

**Keywords:** Short-term exposure, Air pollution, Case-crossover study, Mortality, Kidney diseases, China

## Abstract

**Background:**

Long-term exposure to air pollution has been associated with the onset and progression of kidney diseases, but the association between short-term exposure to air pollution and mortality of kidney diseases has not yet been reported.

**Methods:**

A nationally representative sample of 101,919 deaths from kidney diseases was collected from the Chinese Center for Disease Control and Prevention from 2015 to 2019. A time-stratified case-crossover study was applied to determine the associations. Satellite-based estimates of air pollution were assigned to each case and control day using a bilinear interpolation approach and geo-coded residential addresses. Conditional logistic regression models were constructed to estimate the associations adjusting for nonlinear splines of temperature and relative humidity.

**Results:**

Each 10 µg/m^3^ increment in lag 0–1 mean concentrations of air pollutants was associated with a percent increase in death from kidney disease: 1.33% (95% confidence interval [CI]: 0.57% to 2.1%) for PM_1_, 0.49% (95% CI: 0.10% to 0.88%) for PM_2.5_, 0.32% (95% CI: 0.08% to 0.57%) for PM_10_, 1.26% (95% CI: 0.29% to 2.24%) for NO_2_, and 2.9% (95% CI: 1.68% to 4.15%) for SO_2_.

**Conclusions:**

Our study suggests that short-term exposure to ambient PM_1_, PM_2.5_, PM_10_, NO_2_, and SO_2_ might be important environmental risk factors for death due to kidney diseases in China.

**Supplementary Information:**

The online version contains supplementary material available at 10.1186/s12916-023-02734-9.

## Background

Epidemiological evidence has shown that both short-term and long-term exposure to air pollution is associated with kidney outcomes [1–7]. A few time-series studies reported positive associations between short-term exposure to air pollution and emergency department visits or hospitalizations for kidney diseases in North American and East Asian populations, and the risk ratios ranged from 1.010 to 1.034 [1–3]. The associations of long-term exposure to air pollution with incident kidney diseases and kidney function decline were examined by several large national cohort studies from North America and China, and the magnitude of effect sizes was larger: the hazard ratios or odds ratios ranged from 1.07 to 1.39. Although these previous studies have reported the positive associations between air pollution and renal outcomes, the relationship between short-term air pollution exposure and mortality of kidney disease has not yet been investigated.

Air pollution, especially those with smaller aerodynamic diameter, may be inhaled into the alveoli, penetrate through biological membranes, and enter the blood stream [8]. People with kidney diseases may be particularly vulnerable to these hazardous pollutants since kidneys filter around 20% of cardiac output, and a decline in kidney function may result in retainment of environmental toxins in the blood [9]. Animal-based experimental studies proposed several biological mechanisms that support the detrimental effects of air pollution exposure on the increased risk of kidney dysfunction and mortality, including inflammation and oxidative stress [10–14]. These biological mechanisms, supported by several large epidemiological studies that also report the risk of increased mortality [15–18], justify the plausibility of testing the short-term effects of air pollution on death from kidney diseases in humans.

Time-stratified case-crossover study design has been widely used to estimate acute effects in air pollution epidemiology [17–19]. Controls in this design were simulated on multiple days prior to and after each case day at the same location. Each case serves as its own control to reduce individual-level time-invariant confounding; environmental variables were the factors that vary over time for each person. Although most kidney diseases are chronic in nature, death is typically acute. Thus, a time-stratified case-crossover design is appropriate in estimating the triggering effects of short-term exposure to air pollution on death due to kidney diseases.

In addition to China having the largest number of chronic kidney disease cases (132.3 million) in the world in 2017 [20], the pollution level is well above the reference guidelines proposed by the World Health Organization (WHO) [21–23]. Not only does investigating the relationship between short-term air pollution exposure and death from kidney diseases in China examine this untested hypothesis, but it also offers insights on the burden of death in a country where both factors are extremely prevalent. In this study, we collected a national sample of 101,919 deaths due to kidney diseases in China from 2015 to 2019. We aim to quantify the association between short-term exposure to air pollution and mortality of kidney diseases using a time-stratified case-crossover study design.

## Methods

### Death from kidney diseases

**Fig. 1:**
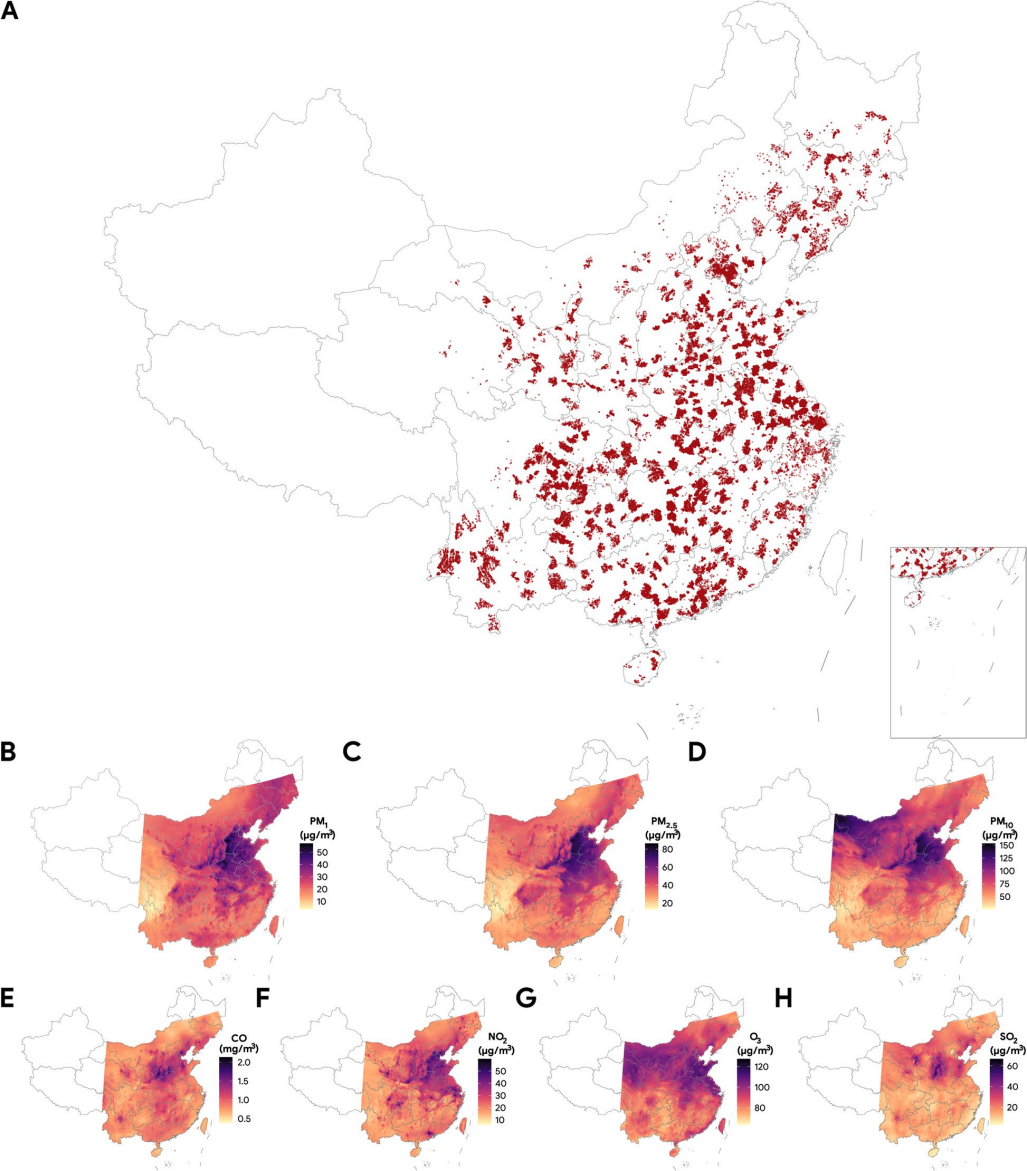
**A** Spatial distribution of a national sample of deaths attributable to kidney diseases in China, from 2015 to 2019 (*N* = 101,919). Each point indicates a death case. **B**–**H** Geographical maps of mean concentrations of ambient air pollution (1B: PM_1_, 1C: PM_2.5_, 1D: PM_10_, 1E: CO, 1F: NO_2_, 1G: O_3_, and 1H: SO_2_) in China from 2015 to 2019

From the National Mortality Surveillance System managed by the Chinese Center for Disease Control and Prevention, we obtained a national sample of deaths due to kidney diseases in China from January 1st, 2015, to December 31st, 2019, using ICD-10 (*International Statistical Classification of Diseases and Related Health Problems, Tenth Revision*) codes “N00” to “N29”. The dates and causes of death were ascertained and completed by certified doctors in the sampling areas. The detailed descriptions of the National Mortality Surveillance System and its application have been published elsewhere [24]. To briefly explain, this system regularly collects causes of death data from 605 counties or districts located in 31 municipalities, provinces, and autonomous regions in mainland China based on multi-stage stratified sampling, covering over 300 million residents (24% of the total population). Data collected by this system have been shown to be representative at the national and provincial levels and have been widely used in formulating national health policies and assessing the burden of disease in China [25]. In Western and Northeastern areas (Xinjiang, Tibet, Qinghai, and northern Heilongjiang) where the population was sparse and very few monitoring stations were available for exposure estimation [26], air pollutants estimates were less robust and the sample size contributed to a small portion of the full sample, so samples in these areas were excluded from the analyses (Fig. 1). The data cleaning steps yielded a final analytical sample of 101,919 deaths from kidney diseases between 2015 and 2019.

### Air pollution data

We used the 10 km × 10 km grid ChinaHighAirPollutants (CHAP) daily data set (available at https://weijing-rs.github.io/product.html) to measure short-term exposure to pollutants [26–31]. CHAP is a long-term, full-coverage, high-resolution, high-quality, and ground-level gridded air pollutant data source. It constructs a combination of advanced satellite remote sensing and space–time models, achieving high cross-validation coefficients of determination of 0.8-0.92 and low root mean square errors compared to the data collected by ground stations [26–31]. This CHAP data set provides daily concentrations of PM_1_, PM_2.5_, PM_10_, O_3_, NO_2_, SO_2_, and CO in China from 2015 to 2019. To reiterate, the air pollutant data for Western and Northeastern China were not available and were not included in the study to ensure the stability and robustness of exposure data. The concentrations of PM_1_, PM_2.5_, PM_10_, NO_2_, SO_2_, and CO were measured as daily average concentrations, while O_3_ was measured as maximum 8-h averages.

Daily averages of meteorological variables (temperature and relative humidity) at 10 km × 10 km grid resolution were obtained from the fifth generation of European ReAnalysis (ERA5)-Land reanalysis data set [32]. The ERA-5 is a high-resolution and long-span dataset for meteorological variables estimated using modern land surface modeling techniques, covering all Chinese regions without missing data from 2015 to 2019.

### Exposure assessment

We used a two-stage assessment strategy to measure the air pollutants and meteorological variables for case and control days, as we have described in our previous studies [33–35]. In the first stage, we geocoded the latitudes and longitudes of residential addresses for each case using the application programming interface provided by *amap* (also known as Gaode map) [36], a leading mapping, navigation, and location-based service provider in China. In the second stage, we used a bilinear interpolation algorithm to estimate the exposure to air pollutants and meteorological variables [37]. This algorithm enhanced the spatial resolution of environmental variables at a specific location by calculating a weighted average of the nearest four grids. The closer the grids of air pollutants and meteorological data were to the location of the cases, the larger the weights applied to the grids. In locations where fewer than four grids were available, we used all the available grids and re-distributed these weights so that the weights sum up to one. These locations accounted for no more than 1% of our data and had at least one measure of the exposure, so we decided to include them in our final analyses.

### Time-stratified case-crossover study design

We determined the association between short-term exposure to air pollutants and the mortality of kidney diseases by adopting a time-stratified case-crossover design [17–19, 38]. This design has the feature that each case serves as its own control by measuring the concentration of exposure on the control days (typically weeks) prior to or after the case day. This study design resembles a classic matched case–control study design, and it accounts for potential confounders that are constant on the case and control days, which are often individual-level time-invariant characteristics. The estimates yielded from a time-stratified case-crossover design therefore represent the acute triggering effect of short-term exposure to air pollution on the outcome.

In this study, we defined each death date as the case day, while the control days were set up to be the days on the same year, month, and day of week as the case day and at the same location. In time-stratified case-crossover designs, each case day typically has three to four control days on the same day of week within the same month [39]. Additional file 1: Fig. S1 presents an example calendar plot of the time-stratified case-crossover design. If a subject died on the first Wednesday of May 2018 (May 2, 2018; the red tile), for example, May 2, 2018, was defined as the case day and all the other Wednesdays in the same month (May 9, 16, 23, and 30, 2018; the blue tiles) were defined as the control days. Using this methodology, 345,926 control days were matched to the 101,919 death cases in this study, yielding a total sample of 447,845 observation days. These matched control days were set up to offset the effects of time trend, seasonality, and day of week.

### Statistical analysis

In the main models, we estimated the odds ratios of death from kidney diseases associated with each 10 µg/m^3^ increase in lag 0–1 of PM_1_, PM_2.5_, PM_10_, O_3_, NO_2_, and SO_2_, as well as 1 mg/m^3^ increase in lag 0–1 of CO using conditional logistic regressions to account for the characteristics of the matched case control study design [19, 38, 40]. To characterize the concentration–response curves of short-term exposure to air pollution and risk of death from kidney diseases, we constructed natural cubic splines (degrees of freedom = 4) in the conditional logistic regression models and plotted the marginal-effect concentration–response curves. Unadjusted conditional logistic regression models accounted for case clustering using patient identification number as strata. Since the exposure conditions for the same individual were switched on the same day of different weeks in the same month (Additional file 1: Fig. S1), the varying exposures nested within the same individuals resemble a matched case–control design and cause the issue of case clustering. Fully adjusted conditional logistic regression models accounted for five-day moving averages (lag 0–4) of daily temperature and relative humidity prior to the date of death by including them as natural restricted cubic splines (degrees of freedom = 4), and case clustering. The lag periods for temperature and relative humidity were chosen based on Bayesian information criterion of the models, and models using lag 0–4 of temperature and relative humidity showed the lowest Bayesian information criterion and the best model fit (Additional file 1: Fig. S2). Since the case-crossover design already accounted for individual-level time-invariant characteristics, individual-level unchanging variables (such as age, sex, and education) were not included in the models. Unadjusted and adjusted percent changes in death from kidney diseases were reported as (odds ratio − 1) × 100% since the odds ratios were very small [17].

### Stratified analyses

We also repeated the conditional logistic regression models after stratifying by several demographic and socioeconomic factors [41–43]: age (< 60, 60–69, 70–79, > 79), sex (men and women), education (< high school and ≥ high school), marriage (married, widowed, and divorced/unmarried), occupation (retired, farmers, other), disease subtype (acute kidney failure, unspecified kidney failure, chronic kidney disease, glomerular diseases, and other kidney diseases), season (cold and warm), year of death (2015, 2016, 2017, 2018, and 2019), and region (Central China, East China, North China, Northeast China, Northwest China, South China, and Southwest China). A map of the seven geographical regions of China is shown in Additional file 1: Fig. S3. Warm seasons were defined as from May to October, while cold seasons were defined as from November to April of the next year. Differences across subgroups were examined by testing the joint significance of the interaction terms between the air pollutant and the subgroup categorical variable using analysis of variance tests.

### Sensitivity analyses

We conducted multiple sensitivity analyses to test the robustness of the results. First, we included different lag periods (lag 0 to lag 3 and lag 0–1 to lag 0–3) of the air pollutants and re-estimated the relative percent changes. Second, we conducted two-pollutant models for each of the seven pollutants in the same model while excluding those models with potential multi-collinearity issues (Pearson correlation coefficient of two pollutants above 0.8).

Missing data were not imputed and were excluded prior to statistical analyses. All p-values were reported two-sided, and a *p*-value smaller than 0.05 or outside the 95% confidence interval (95% CI) excluding null were considered statistically significant. All data management, cleaning, modeling, and visualization were conducted in the Statistical computing environment R 4.1.1 [44]. The study was approved by the institutional review board of Sun Yat-sen University.

## Results

### Sample characteristics and air pollutants

Among the 101,919 deaths from kidney diseases, the median age was 71 (interquartile range [IQR], 59.1 to 80.5) years, 57.9% were men, 72.8% were married, 89.5% received education of lower than high school, and 66.5% were farmers (Table 1). The number of deaths was relatively evenly distributed in across years. The top four regions with the highest number of death cases were East China (25.7%), Southwest China (19.5%), Central China (18.5%), and South China (13.7%). A map of the geographical distribution of deaths is shown in Fig. 1A, where each dot represents a case, and darker clusters denote high density. A total of 345,926 control days were matched to the deaths, resulting in an average of 3.4 control days matched to each case day.

**Table 1:** Characteristics of 101,919 deaths from kidney disease in China from 2015 to 2019

Characteristics	Statistics
Age, years, age (IQR)	71 (59.1–80.5)
Sex	
Men	59,040 (57.9%)
Women	42,879 (42.1%)
Disease	
Total	101,919
Glomerular diseases	62,734 (61.6%)
Acute kidney failure	5756 (5.6%)
Chronic kidney disease	9985 (9.8%)
Unspecified kidney failure	14,563 (14.3%)
Others	8881 (8.7%)
Marriage	
Married	74,245 (72.8%)
Widowed	20,530 (20.1%)
Unmarried/divorced	7144 (7.0%)
Education	
< High school	91,194 (89.5%)
≥ High school	10,725 (10.5%)
Occupation	
Retired	13,653 (13.4%)
Farmer	67,818 (66.5%)
Other	20,448 (20.1%)
Year	
2015	19,424 (19.1%)
2016	19,896 (19.5%)
2017	21,005 (20.6%)
2018	21,051 (20.7%)
2019	20,543 (20.2%)
Region	
Central China	18,851 (18.5%)
East China	26,165 (25.7%)
North China	8970 (8.8%)
Northeast China	8373 (8.2%)
Northwest China	5743 (5.6%)
South China	13,991(13.7%)
Southwest China	19,826 (19.5%)
Season	
Cold	54,682 (53.7%)
Warm	47,237 (46.3%)

**Fig. 2:**
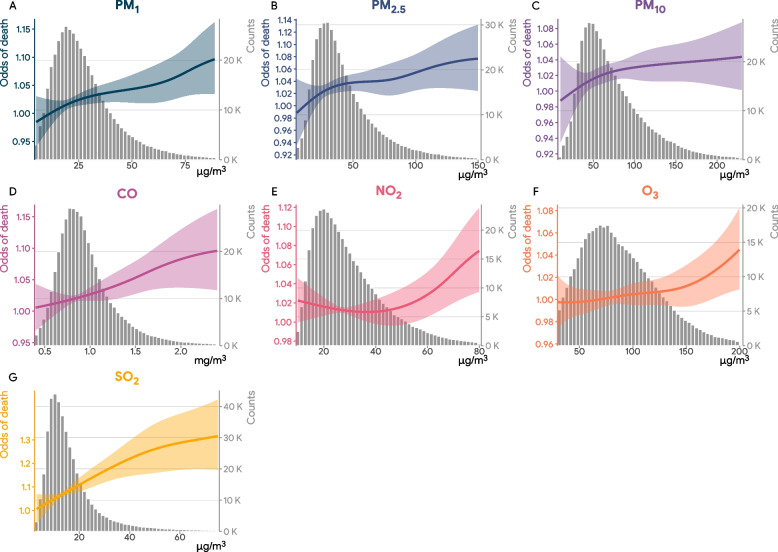
Concentration–response association of short-term exposure to PM_1_, PM_2.5_, PM_10_, CO, NO_2_, O_3_, and SO_2_ with death from kidney diseases in China. The solid lines with shaded regions represent odds of death from kidney diseases and the associated 95% confidence intervals

Table 2 demonstrates the median and interquartile range (IQR) of air pollutants (2-day average, lag 0–1) and meteorological conditions (five-day average, lag 0–4) on all observation days and stratified by control and case days. The concentration of PM_1_, PM_2.5_, PM_10_, NO_2_, and SO_2_ were significantly higher on case days than that of control days, although the magnitude of the difference was small. The overall concentrations of the air pollutants on the observation days were high; for example, the median concentration of PM_2.5_ was 36.36 µg/m^3^ (IQR: 25.2 to 54.52 µg/m^3^) and the median of PM_10_ was 63.32 µg/m^3^ (IQR: 44.28 to 93.84 µg/m^3^). Maps of 5-year mean concentrations of air pollutants in China between 2015 and 2019 are shown in Fig. 2B through Fig. 2H. The temporal trends of case and control days by year and month are shown in Additional file 1: Fig. S4.

**Table 2:** Median (interquartile ranges) of ambient air pollutants and meteorological conditions on control and case days

	**Overall**	**Control days**	**Case days**	*P*-value^*^
	447,845	345,926 (77.2%)	101,919 (22.8%)	
**Particulate matter air pollutants, 2-day moving average (lag 0–1)**				
PM_1_, µg/m^3^	24.68 (17.24–35.41)	24.66 (17.24–35.41)	24.74 (17.24–35.41)	0.003
PM_2.5_, µg/m^3^	36.36 (25.20–54.52)	36.32 (25.2–54.52)	36.50 (25.2–54.50)	0.038
PM_10_, µg/m^3^	63.32 (44.28–93.84)	63.23 (44.28–93.84)	63.63 (44.28–93.84)	0.033
**Gaseous air pollutants, 2-day moving average (lag 0–1)**				
CO, mg/m^3^	0.88 (0.72–1.10)	0.88 (0.72–1.10)	0.88 (0.72–1.10)	0.242
NO_2_, µg/m^3^	26.07 (18.88–36.79)	26.07 (18.88–36.79)	26.08 (18.88–36.79)	0.014
O_3_, µg/m^3^	81.55 (59.38–109.54)	81.55 (59.38–109.54)	81.54 (59.38–109.54)	0.032
SO_2_, µg/m^3^	13.54 (9.60–20.02)	13.53 (9.60–20.02)	13.57 (9.60–20.02)	< 0.001
**Meteorological variables, 5-day moving averages (lag 0–4)**				
Relative humidity, %	72.80 (59.26–81.52)	72.84 (59.26–81.52)	72.68 (59.26–81.52)	0.291
Temperature, Celsius	16.25 (7.92–22.86)	16.28 (7.92–22.86)	16.14 (7.92–22.86)	0.010

^*^The *p*-values were calculated using univariate conditional logistic regression with accounting for the nesting nature of patients in a case-crossover design

### Short-term air pollution exposure associated with increased risk of death from kidney diseases

In the main models, short-term exposure to PM_1_, PM_2.5_, PM_10_, NO_2_, and SO_2_ was associated with a mild but statistically significant increase in the risk of death from kidney diseases in both unadjusted and adjusted models (Table 3). The adjusted relative percent increase in odds of death from kidney disease per 10 µg/m^3^ increase in air pollutants were: 1.33% (95% CI: 0.57% to 2.1%) for PM_1_, 0.49% (95% CI: 0.10% to 0.88%) for PM_2.5_, 0.32% (95% CI: 0.08% to 0.57%) for PM_10_, 1.26% (95% CI: 0.29% to 2.24%) for NO_2_, and 2.9% (95% CI: 1.68% to 4.15%) for SO_2_ (Table 3).

**Table 3:** Short-term ambient air pollution associated with unadjusted and adjusted relative percent increase in odds of death from kidney diseases

Air pollutants^a^	Relative percent increase (95% CI) Unadjusted	*P*-value Unadjusted	Relative percent increase (95% CI) Adjusted†	*P*-value Adjusted†
PM_1_	**1.11 (0.38 to 1.85)**	**0.003**	**1.33 (0.57 to 2.1)**	**0.001**
PM_2.5_	**0.36 (0.013 to 0.73)**	**0.038**	**0.49 (0.10 to 0.88)**	**0.012**
PM_10_	**0.27 (0.04 to 0.51)**	**0.022**	**0.32 (0.08 to 0.57)**	**0.010**
CO	1.94 (− 1.29 to 5.28)	0.242	**3.48 (0.11 to 6.97)**	**0.043**
NO_2_	**1.18 (0.24 to 2.13)**	**0.014**	**1.26 (0.29 to 2.24)**	**0.011**
O_3_	**0.34 (0.03 to 0.65)**	**0.032**	0.34 (− 0.05 to 0.72)	0.084
SO_2_	**3.08 (1.89 to 4.29)**	** < 0.001**	**2.90 (1.68 to 4.15)**	** < 0.001**

PM_1_, PM_2.5_, and PM_10_, particulate matter with diameters less than or equal to 1 µm (2.5 μm for PM_2.5_ and 10 μm for PM_10_); CO, carbon monoxide; NO_2_, nitrogen dioxide; O_3_, ozone; SO_2_, sulfur dioxide; CI, confidence interval

Relative percent increases were reported per 10 µg/m^3^ for PM_1_, PM_2.5_, PM_10_, NO_2_, O_3_, SO_2_ and per 1 mg/m^3^ for CO

^†^Multivariate Conditional logistic regression models adjusted for air pollutants, natural cubic splines of temperature and relative humidity (lag 0-4), and case clustering. Case clustering represents the fact that exposure conditions for the same individual were switched on the same day of different weeks in the same month, and this clustering within the same individuals was accounted for using patient identification number as strata

^a^The pollutants were measured as the moving average of exposure on the day of event and one day prior to the event (lag 0-1)

Figure 2 presents the concentration–response curves of the association between the seven air pollutants and the odds of death from kidney diseases. We observed an increasing but with decreasing gradient trend (a “concave-down” pattern) on the associations of short-term exposure to ambient PM_1_, PM_2.5_, PM_10_, CO, and SO_2_ with odds of death from kidney diseases: the odds of death from kidney diseases increased rapidly at lower concentrations of air pollutants, and it then showed an attenuated increasing trend when the concentration exceeded a threshold. The associations of short-term ambient NO_2_ and O_3_ with odds of death exhibited a slight concave-up relationship.

### Sensitivity analyses

We observed a consistent trend that different lags of short-term exposure (lag 0, lag 1, and MA 01) to PM_1_, PM_2.5_, PM_10_, NO_2_, and SO_2_ were significantly associated with an increased risk of death from kidney diseases (Additional file 1: Fig. S5). The relative percent change estimates were generally significant on the day of the event (lag 0) and the day prior to the event (lag 1), while the relative percent changes were nullified and estimates became insignificant when longer lag periods were used (lag 2 and lag 3).

We further constructed two-pollutant models to test the robustness of the results when the correlation between air pollutants was adjusted for. In view of the high correlation between particulate matter air pollutants (Additional file 1: Fig. S6), the two-pollutant models included one particulate matter air pollutant (PM_1_, PM_2.5_, and PM_10_) and one gaseous pollutant (CO, NO_2_, O_3_, and SO_2_) at a time. The results of two-pollutant models suggest that PM_1_ and SO_2_ remained consistently significant in two-pollutant models (Table 4).

**Table 4:** Short-term ambient air pollution associated with adjusted relative percent increase in odds of death from kidney diseases in two-pollutant models

**Particulate matter air pollution**				
Co-pollutant	Relative percent increase in odds of death from kidney diseases			
	PM_1_	PM_2.5_	PM_10_	
+ CO	**1.43 (0.32 to 2.56)**	0.31 (− 0.24 to 0.86)	0.23 (− 0.08 to 0.53)	
+ NO_2_	0.83 (− 0.23 to 1.89)	0.08 (− 0.43 to 0.58)	0.12 (− 0.18 to 0.42)	
+ O_3_	**1.2 (0.39 to 2.03)**	0.38 (− 0.03 to 0.79)	**0.27 (0.02 to 0.53)**	
+ SO_2_	0.31 (− 0.6 to 1.23)	− 0.04 (− 0.49 to 0.4)	0.05 (− 0.22 to 0.32)	
**Gaseous air pollution**				
Co-pollutant	Relative percent increase in odds of death from kidney diseases			
	CO	NO_2_	O_3_	SO_2_
+ PM_1_	− 1.4 (− 6 to 3.43)	0.71 (− 0.59 to 2.03)	0 (− 0.43 to 0.43)	**2.76 (1.35 to 4.2)**
+ PM_2.5_	1.07 (− 3.59 to 5.95)	**1.27 (0.02 to 2.55)**	0.04 (− 0.39 to 0.47)	**3.07 (1.69 to 4.46)**
+ PM_10_	1.14 (− 2.95 to 5.4)	1.13 (− 0.03 to 2.32)	0.05 (− 0.38 to 0.47)	**2.92 (1.58 to 4.26)**

The pollutants were measured as the average of exposure on the event day and one day prior to the event (lag 0-1)

Conditional logistic regression models adjusted for air pollutants, natural cubic splines of temperature and relative humidity (lag 0-4), and case clustering. Case clustering represents the fact that exposure conditions for the same individual were switched on the same day of different weeks in the same month, and this clustering within the same individuals was accounted for using patient identification number as strata

PM_1_, PM_2.5_, and PM_10_, particulate matter with diameters less than or equal to 1 µm (2.5 μm for PM_2.5_ and 10 μm for PM_10_); CO, carbon monoxide; NO_2_, nitrogen dioxide; O_3_, ozone; SO_2_, sulfur dioxide

Relative percent increases were reported per 10 µg/m^3^ for PM_1_, PM_2.5_, PM_10_, NO_2_, O_3_, SO_2_ and per 1 mg/m^3^ for CO

### Subgroup analyses

**Fig. 3:**
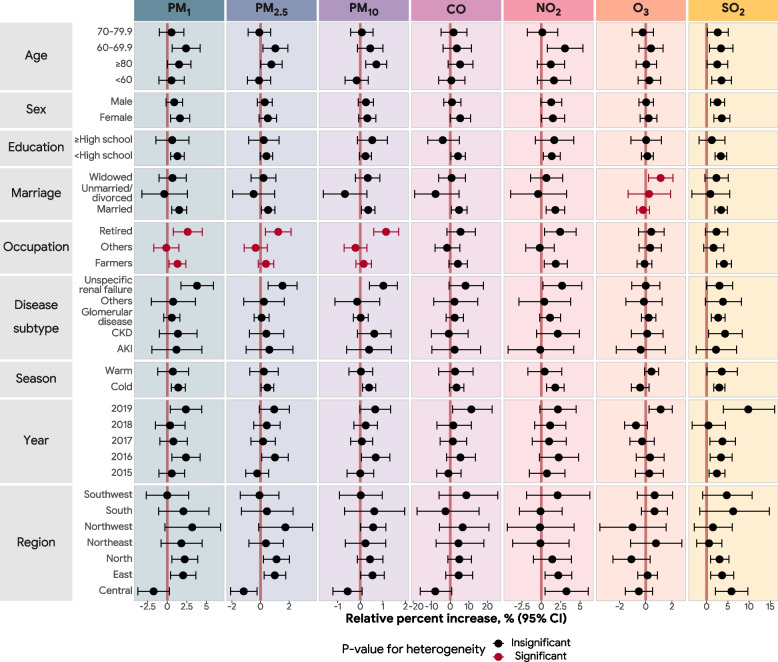
Associations of short-term exposure to air pollution and risk of death from kidney diseases by age, sex, education, marriage, occupation, disease type, season, and region. AKI: acute kidney injury; CKD: chronic kidney disease

Figure 3 and Additional file 1: Table S1 demonstrate the relative percent changes in risk of death from kidney diseases for each air pollutant in stratified analyses by different subgroups. We observed significant differences of percent change estimates across occupation for PM_1_, PM_2.5_, and PM_10_ and across different marital statuses for O_3_. The estimates for PM_1_, PM_2.5_, and PM_10_ were larger among retired residents, and the estimate for O_3_ was larger for widowed individuals. We did not find significant heterogeneity between ambient air pollutants and death from kidney disease in subgroups including age, sex, education, disease subtype, season, year, or region.

## Discussion

In this nationally representative sample of deaths from kidney diseases in China spanning five years, our time-stratified case-crossover study suggests that short-term exposures to PM_1_, PM_2.5_, PM_10_, NO_2_, and SO_2_ were associated with a significantly elevated risk of death, exhibited by a nonlinear concave-down pattern. The results were generally consistent when the exposure was measured at different lag periods and in two-pollutant models.

This was the first nationwide study to examine the relationship between short-term exposure to air pollution and mortality from kidney diseases in China, where both large burden of kidney diseases and air pollution exist. Although air pollution in China has experienced a substantial and steady decline in recent years [45–47], the current level of particulate matter air pollution is still above the 2021 WHO Air Quality Guideline [23]. For example, the medians of particulate matter air pollutants on case days in this study were 34.86 µg/m^3^ for PM_2.5_ and 60.49 µg/m^3^ for PM_10_, both substantially higher than the WHO guideline, in which 24-h pollutants were recommended to be lower than 15 µg/m^3^ and 45 µg/m^3^ respectively. The findings of this study deepen our understanding that elevated air pollution is associated with a small but significant excess in burden of death from kidney disease in China.

Although this epidemiological case-crossover study does not shed mechanistic insights on the link between short-term air pollution exposure and mortality among people with kidney disease, several postulated biological mechanisms may help explain the observed associations in this study. The most widely cited mechanisms are inflammation and oxidative stress, suggested by laboratory experiments and population-based epidemiological evidence [12–14]. Elevation of short-term air pollution may increase pulmonary inflammation and cause damage to distant organs including the kidney, and further trigger death among a vulnerable population [9]. These biological mechanisms need further studies supported by animal-based laboratory evidence or epidemiological studies that delve deeper into the chemical components or different sources of air pollution [9, 22].

Compared to previous cohort studies using long-term exposure to air pollution [48] or aggregated time-series studies [15], the effect estimates in this study were relatively small (< 3% per 10 µg/m^3^ increase); these small estimates are within our expectations given the time-stratified case-crossover design. For example, a large time-stratified case-crossover study in the US Medicare population showed that each 10 µg/m^3^ increase in PM_2.5_ was associated with a 2.3% (95% CI: 1.7% to 3.0%) relative increase in the risk of acute and unspecified renal failure hospital admission [3]. Other time-series studies that investigated associations between short-term exposure to air pollution and emergency department visits for kidney diseases in North American and East Asian population reported relative percent changes ranging from 10% to 3.4% [1, 2]. Another time-stratified case-crossover study in Hubei province of China estimated that each 10 µg/m^3^ increase in PM_2.5_, PM_10_, and NO_2_ was associated with a 4.14% (95% CI: 1.25% to 7.12%), 2.67% (95% CI: 0.80% to 4.57%), and 1.46% (95% CI: 0.76% to 2.17%) increase in odds of myocardial infarction mortality [17]. The magnitude of these effect estimates in previous papers utilizing the same time-stratified case-crossover or time-series study design was comparable to the findings in our study. In addition, we found that the relative percentage increases per 10 µg/m^3^ increment for particulate matter air pollution with smaller particle sizes were greater than those with larger particle sizes were also consistent with findings in several other studies on air pollution and mortality [33, 49].

This study has several strengths. A large nationwide sample of deaths collected from over 600 surveillance sites in China spanning the past five years yields nationally representative estimates on the association between short-term exposure to air pollution and risk of death from kidney diseases. The levels of air pollution, notably particulate matter air pollution, were considerably higher than the WHO-recommended levels, providing us an opportunity to investigate our hypothesis in an ideal setting. Since the time-stratified case-crossover design uses the individual as its own control, it eliminates the possibility of unknown individual-level time-invariant residual confounding including access to renal replacement therapy and genetic risk factors [50, 51]. Compared to other time-series studies that measured air pollution at city levels, our study ascertained air pollution at the individual level using residential latitudes and longitudes, producing more accurate estimates of air pollution.

This study has several limitations. Air pollution was estimated only at residential addresses and did not account for place of employment, and thus, we were unable to obtain a fully comprehensive measurement of air pollution for each case. This may lead to potential exposure misclassification bias, but this is likely a minor issue since the physical activity of patients with kidney problems is limited, and it is unlikely that many individuals were far from their residential addresses for very long. The current CHAP air pollutant data were estimated at 10*10 km grids and had relatively low spatial resolution, this may lead to exposure misclassification and the estimates may be biased towards null. Indoor air pollution subject to cooking, solid fuel use, and smoking is another important source of air pollution that may contribute the death from kidney diseases [52], but this information was unavailable in the study. The exposure source data only measured air pollution as a whole and did not account for the chemical composition of air pollution, and future studies should take advantage of advances in remote sensing technologies and evaluate the effects of chemical components of air pollution [53]. The data collection system included prespecified choices for categorical variables, and some detailed sub-categories may not be collected in the original data (for example, illiteracy for education) [54]. Although the time-stratified case-crossover design eliminates time-invariant factors and we accounted for meteorological variables, it may still be subject to residual confounding caused by unmeasured time-varying factors. The case-crossover design generally has lower statistical power compared to a time-series study design [38], and we were underpowered to detect statistically significant heterogeneity across different subgroups. The findings based on the Chinese population may not be generalizable to other countries due to population dissimilarity and differing levels of air pollution.

## Conclusions

In summary, our results demonstrate a significant association between short-term exposure to PM_1_, PM_2.5_, PM_10_, NO_2_, and SO_2_ and the risk of death from kidney diseases. Policy efforts to reduce air pollution may mitigate the burden of death from kidney diseases in China and globally.

## Supplementary Information

**Additional file 1: Figure S1.** An example calendar heatmap of time-stratified case-crossover study design. **Figure S2.** Bayesian information criterion of models using different lag periods for temperature and relative humidity. **Figure S3.** Spatial distribution of 7 geographical regions of China. **Figure S4.** The number of (A) case days (*N*=101,919) and (B) control days (*N*=345,926) by different months and years in a national sample of deaths due to kidney diseases from 2015 to 2019. **Figure S5.** Different lags and moving averages (MAs) of short-term exposure to air pollution associated with relative percent increases in death from kidney diseases. **Figure S6.** Pairwise Pearson correlation coefficients between the air pollutants. **Table S1.** Association of short-term exposure to air pollution and risk of death from kidney-related deaths by age, sex, education, marriage, occupation, season, and disease type.

## Data Availability

Data may be available upon request.

## Abbreviations

AKI: Acute kidney injury
CHAP: China High Air Pollutants
CI: Confidence interval
CKD: Chronic kidney disease
ERA5: The fifth generation of European ReAnalysis
ICD-10: International Statistical Classification of Diseases and Related Health Problems, Tenth Revision
IQR: Interquartile range
WHO: The World Health Organization
