# Supplementary material for: Short-term air pollution exposure associated with death from kidney diseases: a nationwide time-stratified case-crossover study in China from 2015 to 2019
Source: BMC Med. 2023 Jan 24;21:32. doi: 10.1186/s12916-023-02734-9 (PMC9875429; doi:10.1186/s12916-023-02734-9)
Supplement: Supplementary file 1 — Additional file 1: Figure S1. An example calendar heatmap of time-stratified case-crossover study design. Figure S2. Bayesian information criterion of models using different lag periods for temperature and relative humidity. Figure S3. Spatial distribution of 7 geographical regions of China. Figure S4. The number of (A) case days (N=101,919) and (B) control days (N=345,926) by different months and years in a national sample of deaths due to kidney diseases from 2015 to 2019. Figure S5. Different lags and moving averages (MAs) of short-term exposure to air pollution associated with relative percent increases in death from kidney diseases. Figure S6. Pairwise Pearson correlation coefficients between the air pollutants. Table S1. Association of short-term exposure to air pollution and risk of death from kidney-related deaths by age, sex, education, marriage, occupation, season, and disease type. [file 12916_2023_2734_MOESM1_ESM.docx]

**Supplemental materials**

**Short-term air pollution exposure associated with death from kidney diseases: a nationwide time-stratified case crossover study in China from 2015 to 2019**

**Table of Contents**

| **Content** | **Page** |
| --- | --- |
| **Figure S1**. An example calendar heatmap of time-stratified case crossover study design. The example month is May 2018, the example case day (the red tile) is May 2, 2018, and the example control days (blue tiles) are May 9, 16, 23, and 30, 2018. | 2 |
| **Figure S2**. Bayesian information criterion of models using different lag periods for temperature and relative humidity. | 3 |
| **Figure S3** Spatial distribution of 7 geographical regions of China (Central China, East China, North China, Northeast China, Northwest China, South China, and Southwest China). | 4 |
| **Figure S4**. The number of (A) case days (N=101,919) and (B) control days (N=345,926) by different months and years in a national sample of deaths due to kidney diseases from 2015 to 2019. | 5 |
| **Figure S5**. Different lags and moving averages (MAs) of short-term exposure to air pollution associated with relative percent increases in death from kidney diseases. | 6 |
| **Figure S6.** Pairwise Pearson correlation coefficients between the air pollutants. | 7 |
| **Table S1.** Association of short-term exposure to air pollution and risk of death from kidney-related deaths by age, sex, education, marriage, occupation, season, and disease type. | 8-9 |


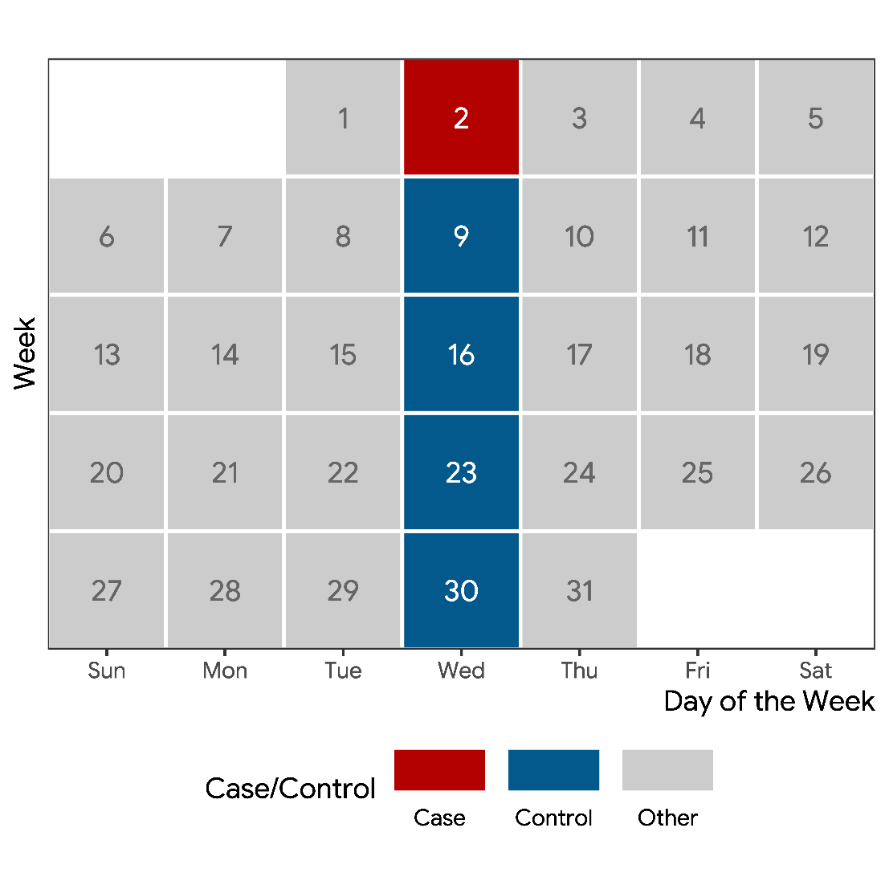


**Figure S1**. An example calendar heatmap of time-stratified case crossover study design. The example month is May 2018, the example case day (the red tile) is May 2, 2018, and the example control days (blue tiles) are May 9, 16, 23, and 30, 2018.


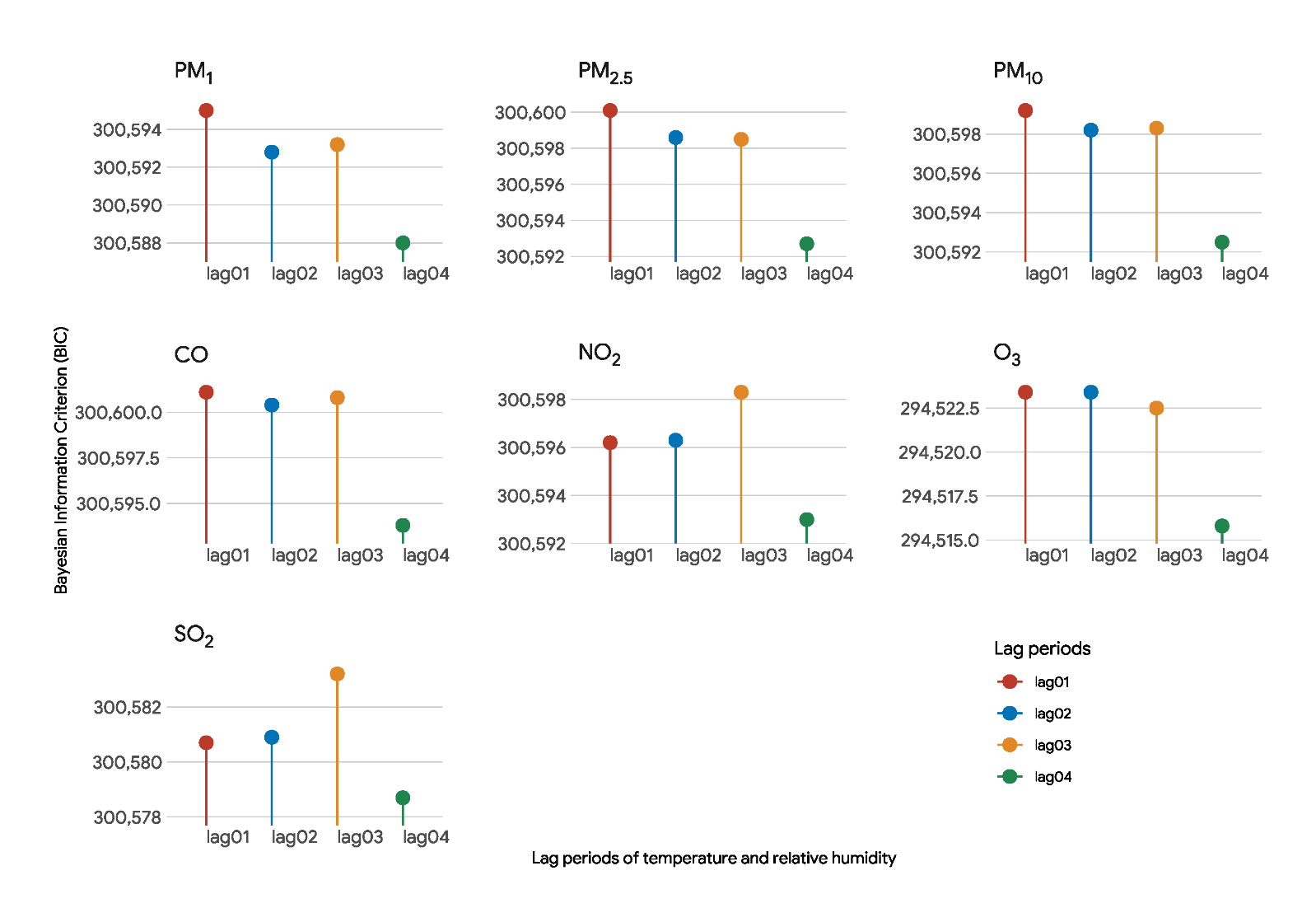


**Figure S2**. Bayesian information criterion of models using different lag periods for temperature and relative humidity.

**
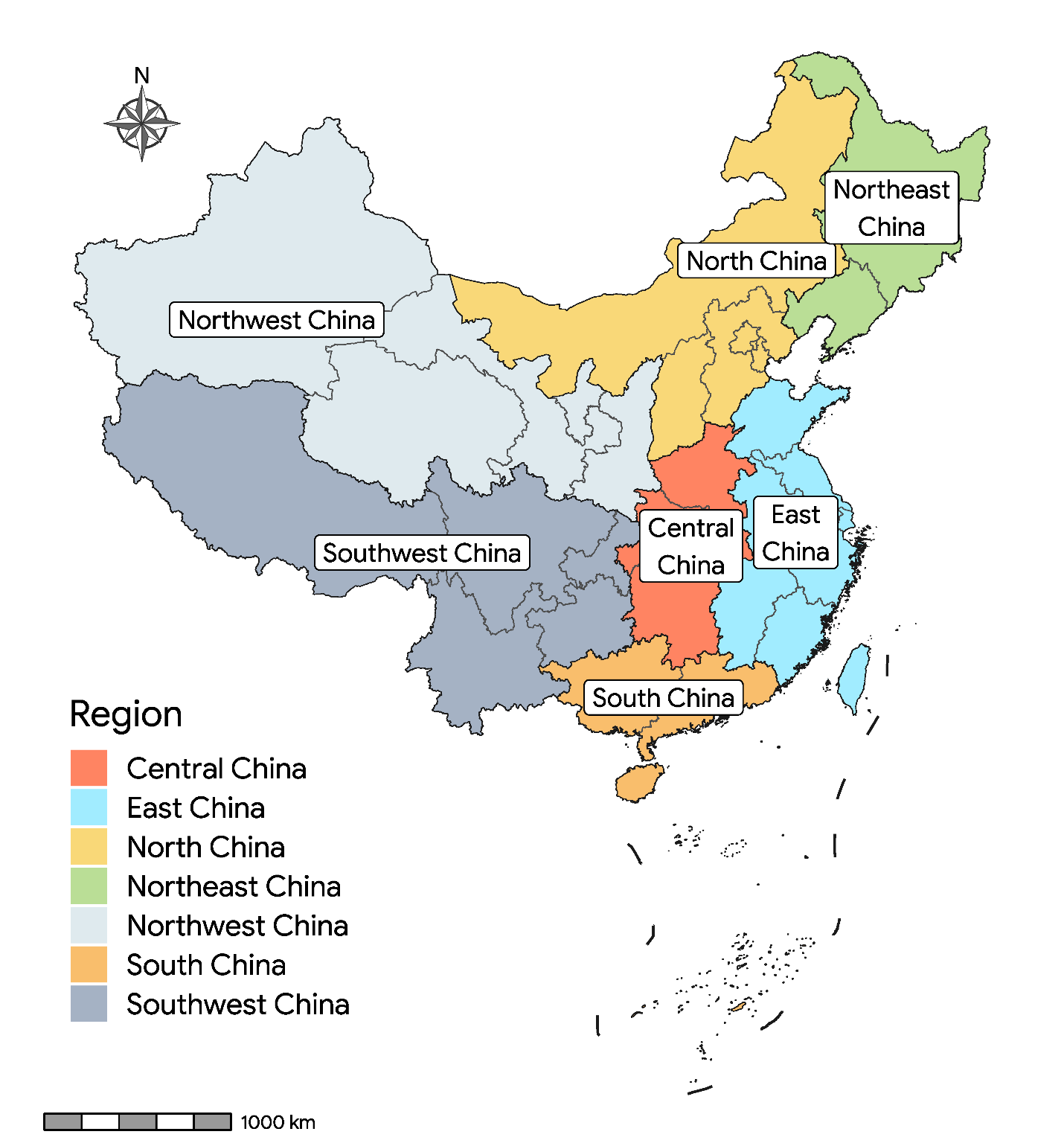
**

**Figure S3** Spatial distribution of 7 geographical regions of China (Central China, East China, North China, Northeast China, Northwest China, South China, and Southwest China).


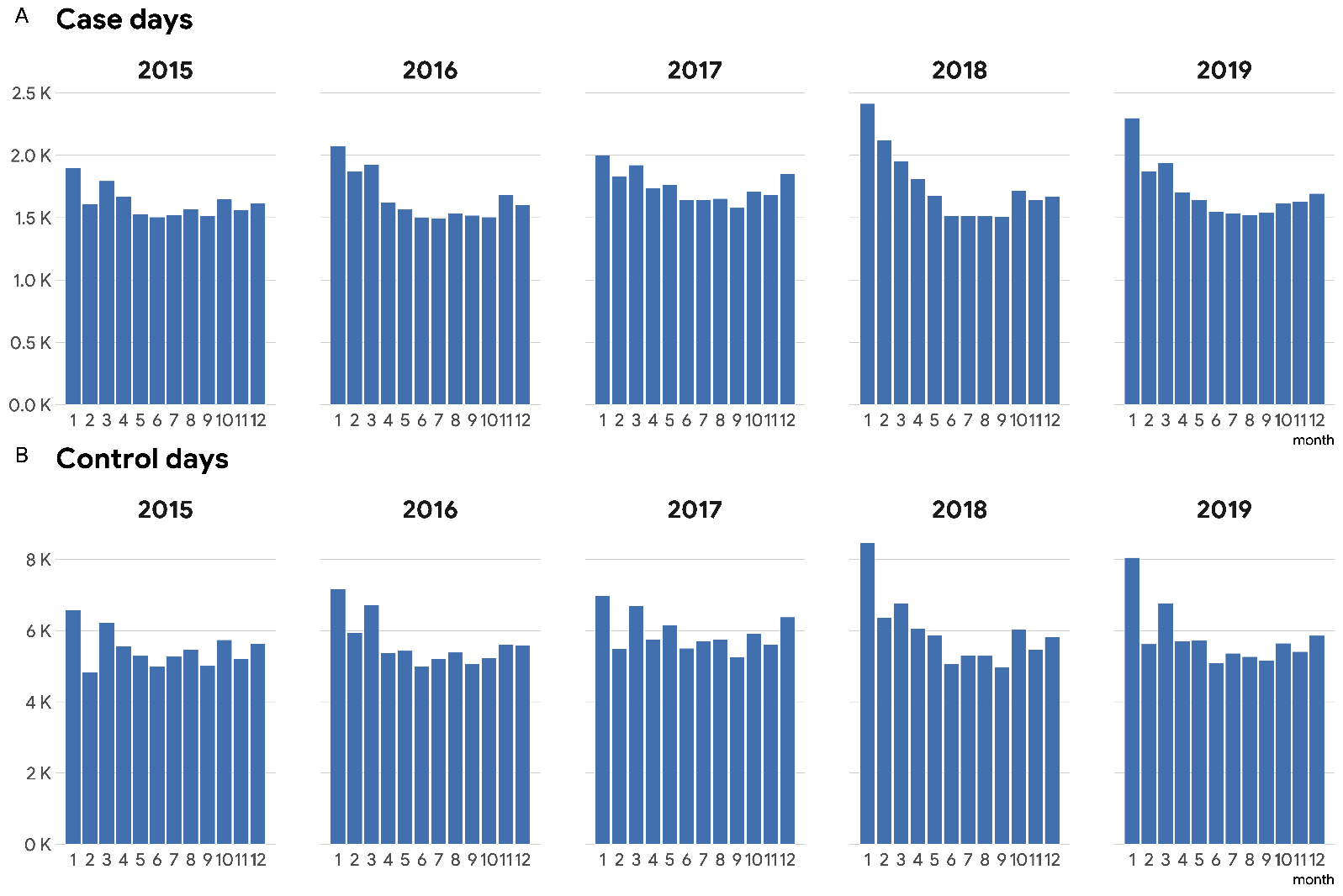


**Figure S4**. The number of (A) case days (N=101,919) and (B) control days (N=345,926) by different months and years in a national sample of deaths due to kidney diseases from 2015 to 2019.

**
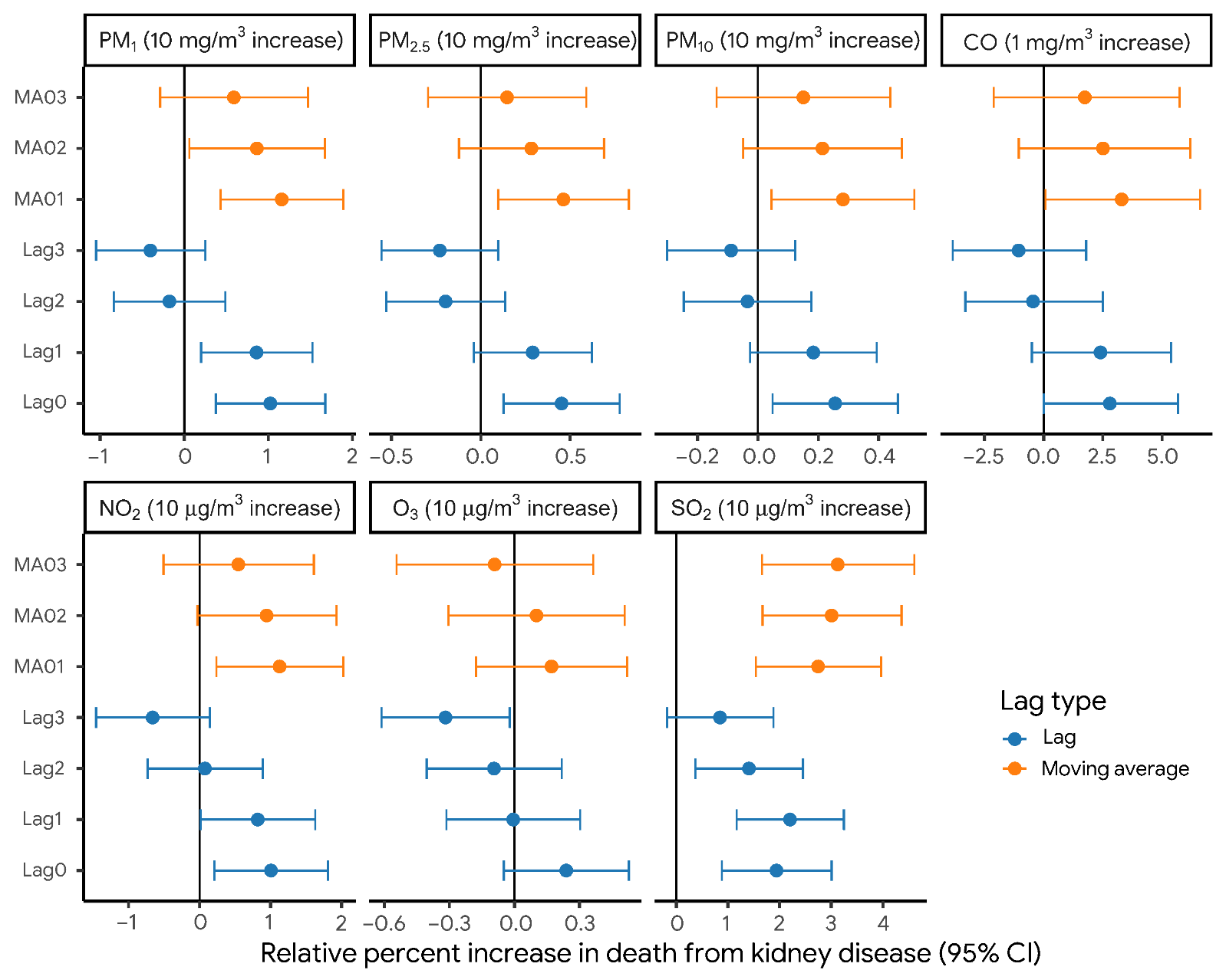
 Figure S5**. Different lags and moving averages (MAs) of short-term exposure to air pollution associated with relative percent increases in death from kidney diseases.


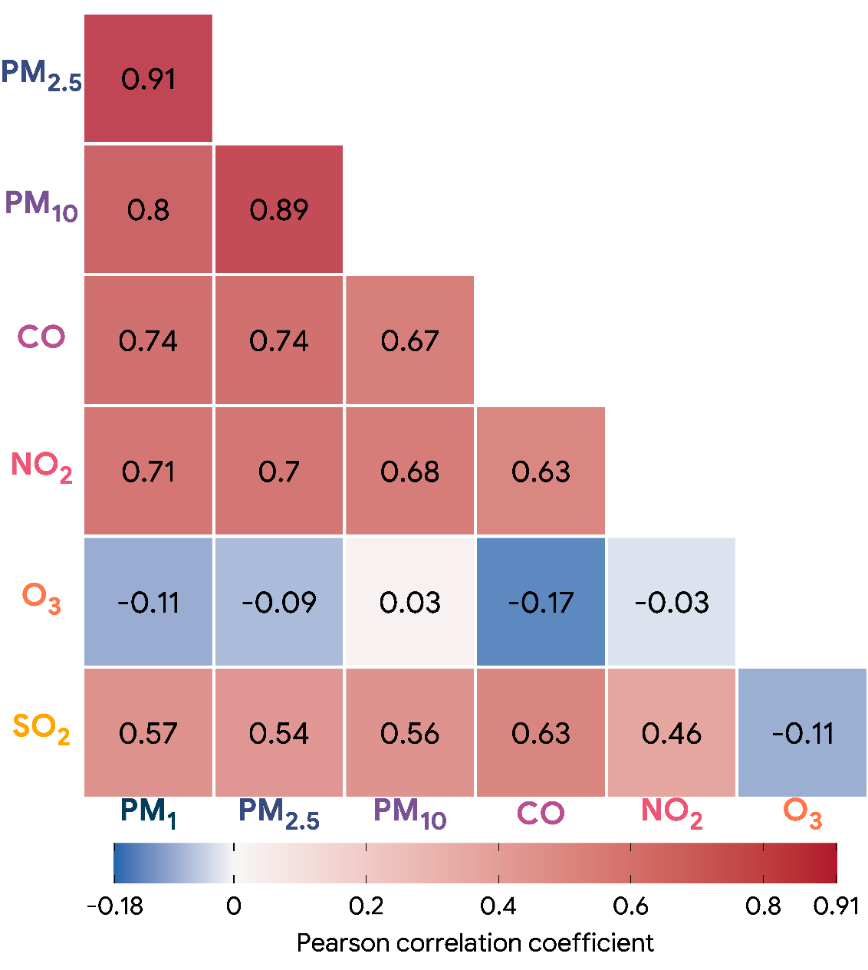


**Figure S6.** Pairwise Pearson correlation coefficients between the air pollutants.

**Table S1** Association of short-term exposure to air pollution and risk of death from kidney-related deaths by age, sex, education, marriage, occupation, season, and disease types

|  | **PM_1_** | **PM_2.5_** | **PM_10_** | **CO** | **NO_2_** | **O_3_** | **SO_2_** |
| --- | --- | --- | --- | --- | --- | --- | --- |
| **Age** |  |  |  |  |  |  |  |
| P-value for heterogeneity | 0.325 | 0.153 | 0.08 | 0.853 | 0.432 | 0.897 | 0.982 |
| <60 | 0.54 (-1.07 to 2.17) | -0.1 (-0.91 to 0.71) | -0.16 (-0.67 to 0.35) | 0.59 (-6.22 to 7.9) | 1.63 (-0.48 to 3.79) | 0.27 (-0.59 to 1.14) | 3.51 (1.2 to 5.88) |
| 60-69 | 2.39 (0.62 to 4.19) | 1.04 (0.17 to 1.92) | 0.44 (-0.12 to 1) | 3.55 (-3.81 to 11.47) | 3.05 (0.78 to 5.37) | 0.4 (-0.53 to 1.33) | 3.4 (0.7 to 6.18) |
| 70-79 | 0.54 (-1.02 to 2.13) | -0.08 (-0.86 to 0.71) | 0.07 (-0.42 to 0.57) | 1.81 (-4.89 to 8.99) | 0.14 (-1.79 to 2.12) | -0.23 (-1.05 to 0.6) | 2.62 (0.17 to 5.13) |
| >80 | 1.5 (0.03 to 3) | 0.76 (0.02 to 1.51) | 0.71 (0.24 to 1.17) | 5.3 (-1.13 to 12.14) | 1.23 (-0.5 to 2.99) | 0.04 (-0.74 to 0.82) | 2.51 (0.11 to 4.95) |
| **Sex** |  |  |  |  |  |  |  |
| P-value for heterogeneity | 0.1 | 0.218 | 0.428 | 0.084 | 0.42 | 0.398 | 0.313 |
| Men | 0.89 (-0.16 to 1.95) | 0.3 (-0.22 to 0.83) | 0.25 (-0.09 to 0.58) | 0.95 (-3.57 to 5.68) | 1.28 (-0.04 to 2.62) | 0.03 (-0.53 to 0.59) | 2.55 (0.92 to 4.22) |
| Women | 1.62 (0.4 to 2.85) | 0.51 (-0.1 to 1.12) | 0.31 (-0.07 to 0.69) | 5.36 (0.08 to 10.93) | 1.5 (0.01 to 3.02) | 0.22 (-0.43 to 0.86) | 3.58 (1.71 to 5.48) |
| **Education** |  |  |  |  |  |  |  |
| P-value for heterogeneity | 0.202 | 0.356 | 0.838 | 0.072 | 0.785 | 0.22 | 0.08 |
| Middle school and below | 1.3 (0.43 to 2.17) | 0.42 (-0.01 to 0.85) | 0.23 (-0.04 to 0.5) | 4.17 (0.38 to 8.11) | 1.35 (0.26 to 2.45) | 0.13 (-0.33 to 0.58) | 3.36 (2.01 to 4.74) |
| High school and above | 0.63 (-1.46 to 2.76) | 0.23 (-0.82 to 1.3) | 0.53 (-0.12 to 1.2) | -4.03 (-12.15 to 4.83) | 1.67 (-0.77 to 4.17) | 0.03 (-1.14 to 1.21) | 1.24 (-1.74 to 4.3) |
| **Marriage** |  |  |  |  |  |  |  |
| P-value for heterogeneity | 0.685 | 0.597 | 0.184 | 0.273 | 0.539 | **0.014** | 0.637 |
| Widowed | 0.67 (-1.04 to 2.42) | 0.21 (-0.66 to 1.08) | 0.33 (-0.21 to 0.87) | 0.72 (-6.25 to 8.2) | 0.66 (-1.4 to 2.77) | **1.14 (0.21 to 2.08)** | 2.32 (-0.4 to 5.11) |
| Married | 1.52 (0.57 to 2.48) | 0.53 (0.06 to 1.01) | 0.35 (0.05 to 0.65) | 4.74 (0.55 to 9.11) | 1.81 (0.61 to 3.02) | **-0.21 (-0.7 to 0.29)** | 3.42 (1.97 to 4.89) |
| Unmarried/divorced | -0.38 (-3.23 to 2.57) | -0.48 (-1.94 to 1) | -0.68 (-1.64 to 0.29) | -7.9 (-19.06 to 4.81) | -0.41 (-3.94 to 3.25) | **0.25 (-1.35 to 1.89)** | 0.9 (-3.46 to 5.46) |
| **Occupation** |  |  |  |  |  |  |  |
| P-value for heterogeneity | **0.018** | **0.014** | **0.001** | 0.108 | 0.059 | 0.423 | 0.137 |
| Retired | **2.6 (0.77 to 4.46)** | **1.24 (0.35 to 2.14)** | **1.14 (0.59 to 1.7)** | 5.54 (-1.84 to 13.47) | 2.43 (0.42 to 4.47) | 0.42 (-0.55 to 1.41) | 2.32 (-0.27 to 4.98) |
| Farmer | **1.3 (0.24 to 2.38)** | **0.38 (-0.15 to 0.92)** | **0.15 (-0.19 to 0.5)** | 4.1 (-0.72 to 9.15) | 1.86 (0.39 to 3.34) | -0.09 (-0.66 to 0.47) | 4.09 (2.35 to 5.87) |
| Other | **-0.13 (-1.73 to 1.49)** | **-0.34 (-1.15 to 0.48)** | **-0.21 (-0.72 to 0.29)** | -1.73 (-8.15 to 5.14) | -0.19 (-2.01 to 1.67) | 0.34 (-0.51 to 1.19) | 1.63 (-0.7 to 4.02) |
| **Season** |  |  |  |  |  |  |  |
| P-value for heterogeneity | 0.789 | 0.877 | 0.269 | 0.82 | 0.255 | 0.131 | 0.982 |
| Cold | 1.4 (0.51 to 2.29) | 0.48 (0.04 to 0.93) | 0.39 (0.1 to 0.68) | 3.36 (-0.43 to 7.29) | 1.8 (0.66 to 2.94) | -0.43 (-1.11 to 0.26) | 2.99 (1.67 to 4.33) |
| Warm | 0.72 (-1.22 to 2.71) | 0.24 (-0.75 to 1.24) | 0.03 (-0.5 to 0.55) | 2.61 (-6.2 to 12.24) | 0.43 (-1.71 to 2.63) | 0.44 (-0.1 to 0.98) | 3.58 (0.07 to 7.21) |
| **Disease type** |  |  |  |  |  |  |  |
| P-value for heterogeneity | 0.214 | 0.23 | 0.057 | 0.83 | 0.719 | 0.964 | 0.932 |
| Chronic kidney disease | 1.36 (-1.02 to 3.8) | 0.41 (-0.77 to 1.62) | 0.62 (-0.12 to 1.36) | -0.78 (-10.17 to 9.59) | 2.12 (-0.62 to 4.93) | 0.11 (-1.1 to 1.33) | 4.35 (0.43 to 8.42) |
| Glomerular diseases | 0.58 (-0.46 to 1.62) | 0.08 (-0.44 to 0.6) | 0.02 (-0.31 to 0.35) | 2.37 (-2.22 to 7.17) | 1.13 (-0.2 to 2.47) | 0.22 (-0.33 to 0.78) | 2.73 (1.12 to 4.36) |
| Acute kidney failure | 1.17 (-1.97 to 4.42) | 0.62 (-1 to 2.26) | 0.39 (-0.6 to 1.38) | 2.36 (-9.91 to 16.31) | -0.13 (-4.26 to 4.17) | -0.39 (-2.25 to 1.5) | 2.22 (-2.41 to 7.06) |
| Unspecified kidney failure | 3.79 (1.73 to 5.89) | 1.54 (0.53 to 2.56) | 1.02 (0.41 to 1.64) | 8.21 (-0.62 to 17.82) | 2.68 (0.22 to 5.21) | 0 (-1.08 to 1.08) | 3.03 (-0.01 to 6.15) |
| Other kidney diseases | 0.75 (-2.04 to 3.61) | 0.24 (-1.17 to 1.67) | -0.14 (-1.12 to 0.86) | 2.29 (-8.85 to 14.8) | 0.39 (-2.89 to 3.78) | -0.14 (-1.52 to 1.25) | 3.87 (-0.3 to 8.22) |
| **Year** |  |  |  |  |  |  |  |
| P-value for heterogeneity | 0.72 | 0.275 | 0.77 | 0.156 | 0.941 | 0.811 | 0.348 |
| 2015 | 0.57 (-1.06 to 2.22) | -0.23 (-1.03 to 0.57) | 0 (-0.58 to 0.59) | -0.98 (-7.32 to 5.8) | 0.73 (-1.53 to 3.04) | 0.27 (-0.79 to 1.34) | 2.43 (0.58 to 4.31) |
| 2016 | 2.38 (0.6 to 4.19) | 1.01 (0.08 to 1.94) | 0.68 (0.05 to 1.31) | 5.49 (-2.02 to 13.59) | 2.24 (-0.24 to 4.78) | 0.33 (-0.72 to 1.4) | 3.39 (0.84 to 6.01) |
| 2017 | 0.78 (-0.94 to 2.54) | 0.18 (-0.67 to 1.04) | 0.07 (-0.42 to 0.55) | 1.42 (-5.46 to 8.8) | 1.01 (-1.15 to 3.21) | -0.28 (-1.21 to 0.66) | 3.74 (0.77 to 6.79) |
| 2018 | 0.36 (-1.51 to 2.27) | 0.44 (-0.47 to 1.37) | 0.24 (-0.28 to 0.76) | 1.65 (-7.15 to 11.27) | 1.14 (-0.86 to 3.18) | -0.75 (-1.63 to 0.14) | 0.45 (-3.39 to 4.45) |
| 2019 | 2.37 (0.38 to 4.4) | 0.96 (-0.08 to 2.02) | 0.66 (-0.02 to 1.34) | 11.33 (1.19 to 22.49) | 2.14 (-0.17 to 4.49) | 1.14 (0.26 to 2.03) | 9.81 (3.93 to 16.02) |
| **Region** |  |  |  |  |  |  |  |
| P-value for heterogeneity | 0.324 | 0.077 | 0.41 | 0.532 | 0.345 | 0.187 | 0.426 |
| Central China | -1.74 (-3.73 to 0.29) | -1.17 (-2.09 to -0.24) | -0.56 (-1.21 to 0.08) | -8.05 (-16.09 to 0.76) | 3.24 (0.5 to 6.06) | -0.53 (-1.56 to 0.51) | 5.86 (2.12 to 9.74) |
| East China | 2.02 (0.41 to 3.65) | 1 (0.25 to 1.76) | 0.54 (0.02 to 1.06) | 4.54 (-2.48 to 12.06) | 2.19 (0.48 to 3.92) | 0.14 (-0.61 to 0.9) | 3.65 (0.98 to 6.39) |
| North China | 2.22 (0.59 to 3.88) | 1.12 (0.21 to 2.03) | 0.43 (-0.13 to 1) | 4.87 (-1.25 to 11.38) | 1.42 (-1 to 3.89) | -1.1 (-2.51 to 0.33) | 3.05 (0.9 to 5.26) |
| Northeast China | 1.8 (-0.78 to 4.45) | 0.38 (-0.81 to 1.6) | 0.23 (-0.66 to 1.12) | 4.42 (-7.63 to 18.04) | -0.11 (-3.67 to 3.59) | 0.78 (-1.17 to 2.77) | 0.58 (-2.34 to 3.59) |
| Northwest China | 3.18 (-0.3 to 6.78) | 1.74 (-0.12 to 3.64) | 0.57 (0.01 to 1.13) | 6.69 (-5.79 to 20.82) | -0.16 (-4.37 to 4.23) | -1.01 (-3.51 to 1.56) | 1.5 (-2.87 to 6.06) |
| South China | 2.06 (-1.09 to 5.3) | 0.45 (-1.33 to 2.28) | 0.62 (-0.7 to 1.96) | -2.55 (-17.8 to 15.52) | -0.1 (-2.8 to 2.67) | 0.67 (-0.32 to 1.66) | 6.27 (-1.6 to 14.76) |
| Southwest China | -0.01 (-2.68 to 2.73) | -0.07 (-1.41 to 1.29) | 0.02 (-0.92 to 0.97) | 8.64 (-5.95 to 25.51) | 2.1 (-1.89 to 6.25) | 0.68 (-0.65 to 2.04) | 4.75 (-0.91 to 10.73) |

.
